# Octreotide Does Not Inhibit Proliferation in Five Neuroendocrine Tumor Cell Lines

**DOI:** 10.3389/fendo.2018.00146

**Published:** 2018-04-06

**Authors:** Samantha Exner, Vikas Prasad, Bertram Wiedenmann, Carsten Grötzinger

**Affiliations:** ^1^Department of Hepatology and Gastroenterology, Charité – Universitätsmedizin Berlin, Berlin, Germany; ^2^Partner Site Berlin, German Cancer Consortium (DKTK), German Cancer Research Center (DKFZ), Heidelberg, Germany; ^3^Department of Nuclear Medicine, Charité – Universitätsmedizin Berlin, Berlin, Germany; ^4^Department of Nuclear Medicine, Universitätsklinikum Ulm, Ulm, Germany

**Keywords:** neuroendocrine tumor, somatostatin receptor, somatostatin analog, cell line, octreotide, radiation sensitivity, peptide receptor radionuclide therapy, expression

## Abstract

Somatostatin analogs (SSA) are well-established antisecretory drugs in functionally active neuroendocrine tumors (NET). Two placebo-controlled trials have recently demonstrated significant improvement of progression-free survival under SSA treatment. Furthermore, somatostatin receptor (SSTR) overexpression in NET has also been utilized for diagnostic imaging and peptide receptor radionuclide therapy (PRRT). However, PRRT in NET is associated mostly with partial and minor remission, while other radionuclide therapies reach complete remissions in up to 75% of cases. This study assessed a potential radiosensitizing effect of SSA treatment in five established NET cell line models: BON, QGP-1, LCC-18, H727, and UMC-11. Irradiation was found to significantly inhibit proliferation, while no additional effect by octreotide treatment was observed. Intriguingly, no impact of SSA treatment alone was found in any of these NET cell lines when systematically analyzing cell viability, proliferation, and cell cycle distribution. Investigation of the causes for this octreotide resistance led to demonstration of low octreotide binding and scarce SSTR, specifically SSTR2 expression as compared to levels found in human NETs. The resistance toward SSA treatment in viability and proliferation assays could not be overcome by re-expression of SSTR2 in two of the cell lines. These results provide systematic evidence for a lack of authentic, tumor-like SSTR expression, and function in five frequently used NET cell line models and point to the need for more physiologic tumor model systems.

## Introduction

Somatostatin analogs (SSAs) are well-established antisecretory drugs with a favorable safety profile that have been used as first-line treatment to control hormone hypersecretion in functionally active neuroendocrine tumors (NET) for more than 30 years (1, 2). Depot formulations of SSAs, lanreotide autogel, and long-acting repeatable (LAR) octreotide appear to be equally effective and well tolerated (3). The relevance of SSAs in antiproliferative treatment has been a matter of debate for the past two decades. However, two placebo-controlled trials have recently demonstrated significant improvement of progression-free survival under SSA treatment (4, 5). The PROMID trial was a placebo-controlled, double-blind, randomized study evaluating the effect of octreotide LAR on tumor growth in patients with metastatic, well-differentiated midgut NET. The study showed that octreotide LAR significantly prolonged time to progression as compared with placebo (median time to tumor progression 14.3 and 6 months, respectively) (4). More recently, another randomized, double-blind, placebo-controlled trial evaluated the effect of lanreotide in patients with metastatic, well- or moderately differentiated, non-functional enteropancreatic NET (the CLARINET trial). It showed that lanreotide treatment resulted in a significantly extended progression-free survival as compared to placebo (median progression-free survival not reached vs. 18 months) (5). As in the PROMID trial, this did not translate to increases in overall survival.

Somatostatin receptors (SSTR) comprise five closely related G protein-coupled receptors with high affinity to their cognate peptides, somatostatin-14 and somatostatin-28 (6). SSTRs were cloned during the late 1990s from various species such as rat, mouse, and human. The five SSTR subtypes showed a high degree of evolutionary conservation (7). In overexpression systems employing cell lines such as CHO, COS, and HEK293, SSTRs have shown to signal *via* the G_i_ pathway, thus lowering intracellular cAMP levels. In addition, various other signaling pathways and antiproliferative mechanisms for SSTRs have been described in these cellular models (8).

The overexpression of SSTRs in NETs as the basis for therapeutic approaches with SSAs had first been described during the 1980s. Autoradiographic detection of SSTRs using radioiodinated SSAs and *in situ* hybridization were used at that time to demonstrate high overexpression in tumors with little background in other organs (9). With the availability of specific antibodies for SSTRs, these results could be confirmed using immunohistochemistry (10–12). Apart from direct pharmacological intervention for symptom control and antiproliferative action, SSTR overexpression in NET has also been utilized for targeted imaging and peptide receptor radionuclide therapy (PRRT) (13–16). PRRT using peptide conjugates such as DOTATOC and DOTATATE has evolved to be a major treatment option in the management of NETs and is associated mostly with partial and minor remission with objective response rates ranging from 4 to 30% (17). While other radionuclide treatments (radioiodine therapy for medullary thyroid cancer, radioimmunotherapy in B-cell non-Hodgkin’s lymphoma) reach high complete remission rates, drugs that act as radiosensitizers may improve PRRT success rates. Whether SSAs could sensitize tumors to radionuclide therapy is also of current clinical interest, as the first randomized multicenter PRRT trial in NET (NETTER-1) recently evaluated SSA treatment alone vs. a combination of PRRT plus SSA (18, 19).

This study was conceived to assess a potential radiosensitizing effect of SSA treatment in five established NET cell lines. The consequence of octreotide treatment before irradiation vs. irradiation only was to be determined. Further on, the impact of SSA treatment alone on these NET cell lines was to be examined systematically by analyzing cell viability (metabolic activity), proliferation (cell number), and cell cycle distribution. Additional aims were to evaluate somatostatin binding and expression levels of all five SSTR subtypes in these cell lines. Finally, the impact of SSTR2 overexpression in two of the cell lines on viability under octreotide treatment was to be analyzed.

## Materials and Methods

If not indicated otherwise, cell culture reagents were obtained from Biochrom AG (Berlin, Germany) and chemicals, enzymes or dyes from Sigma Aldrich (Deisenhofen, Germany).

### Cell Culture

The human neuroendocrine cell lines BON and QGP-1 (pancreatic), LCC-18 (colonic), and H727 and UMC-11 (pulmonary) were cultured in RPMI 1640 containing 10% fetal calf serum in a humidified atmosphere at 37°C with 5% CO_2_. BON cells were a kind gift of Courtney Townsend (University of Texas, Galveston, TX, USA). LCC-18 cells were kindly donated by Kjell Öberg (University of Uppsala, Sweden). QGP-1 were obtained from the Japanese Collection of Research Bioresources, and H727 and UMC-11 from ATCC (Manassas, VA, USA). Cells were cultured for no longer than 15 passages. All human cell lines have been authenticated by DSMZ (Braunschweig, Germany) using STR profiling. For QGP-1, UMC-11, and H727, DSMZ confirmed their identity as authentic. Due to the lack of reference profiles for BON and LCC-18, their respective STR profile was characterized as unique and not contaminated with any known cell line. The results of the STR profiling for BON and LCC-18 are provided in the table below.

**Table:** 

	D5	D5’	D13	D13’	D7	D7’	D16	D16’	vWA	vWA’	TH01	TH01’	TPOX	TPOX’	CSF1	CSF1’	Amel	Amel’
BON	9	12	11	12	9	9	10	11	18	19	8.0	8.0	9.0	9.0	10	11	X	Y
LCC-18	14	14	8	11	11	12	10	13	16	19	9.0	9.3	8.0	10.0	11	11	X	X

The recombinant cell lines BON-SSTR2 and QGP-1-SSTR2 were generated by transfecting wild-type BON and QGP-1 cells with pcDNA3.1-huSSTR2 (#SSTR200000, cDNA Resource Center, Bloomsberg, PA, USA; www.cdna.org) and jetPEI transfection reagent (Polyplus-transfection, Illkirch, France). Positive clones were selected by addition of 600 μg/ml G-418 and tested for their SSTR2 expression by radioligand binding assay. RIN-1038 were a kind gift from Jacques Philippe (University of Geneva, Switzerland) and were cultured in RPMI 1640 supplemented with 10% fetal calf serum. RIN-1038-SSTR2-GFP cells were generated by stable transfection (see above) with a corresponding plasmid containing ratSSTR2-GFP. The transfected cell lines were constantly maintained in RPMI 1640 supplemented with 10% fetal calf serum and 400 μg/ml G-418.

### Human Tissues

This study was carried out in accordance with the recommendations and protocol approval by the local ethics committee at Charité—Universitätsmedizin Berlin with written informed consent from all subjects, in accordance with the Declaration of Helsinki. Sample characteristics are summarized in Table S2 in Supplementary Material.

### Reverse Transcription Quantitative Real-Time PCR (RT-qPCR)

Total RNA was isolated from NET cell lines or patient tissues using the RNeasy Mini Kit (Qiagen, Hilden, Germany) according to the manufacturer’s protocol. RNA was treated with DNase I (1 U/μg RNA) prior to reverse transcription with the High Capacity cDNA Reverse Transcription Kit (Applied Biosystems, Waltham, MA, USA) according to the protocol supplied by the manufacturer. Quantitative real-time PCR was performed with SsoFast EvaGreen Supermix (Bio-Rad Laboratories, Hercules, CA, USA), 0.5 µM primer, and 30 ng cDNA in 10 µl total reaction volume on a Bio-Rad CFX96 Real-Time-System. PCR conditions were as follows: 98°C for 30 s, followed by 45 cycles of 98°C for 3 s and 60°C for 30 s. All primers were designed by using Primer3Plus, NCBI Primer-BLAST, and UCSC BLAT software. Primers were manufactured by Tib MolBiol (Berlin, Germany); their sequences are indicated in Table S1 in Supplementary Material. Plotted values were normalized to ALG9 and HPRT1 using the ΔΔCt method (20). Reference genes were validated and chosen using geNorm algorithm by qbase+ software (Biogazelle, Ghent, Belgium).

### Drug and Radiation Treatment

Octreotide was synthesized by peptides & elephants (Hennigsdorf, Germany). For treatment, cells were seeded as desired and incubated overnight. The drug was always applied in medium on top of the wells at twofold concentration for final concentrations between 0.001 nM and 10 µM. For experiments involving radiation, cells were irradiated 24 h after pretreatment using an external ^137^Cs source (GSR D1, Gamma-Service Medical, Leipzig, Germany) at a dose rate of 1 Gy/min and further incubated at 37°C without medium change for the indicated time.

### Cell Viability and Proliferation Assays

Cell lines were seeded in quadruplicates in 96-well plates at a density of 5,000 cells per well and grown overnight. Cells were treated with the indicated concentrations of octreotide in 100 µl medium per well. Metabolic activity and cell number were determined after another 96 h. For this, 100 µl medium containing AlamarBlue™ redox indicator (Thermo Fisher, Waltham, MA, USA) were added on top of each well, incubated for 3–4 h and the resulting fluorescence was measured using an EnVision Multilabel Plate Reader (Perkin Elmer, Waltham, MA, USA). Afterward, the supernatant was removed; cells were fixated with 4% v/v formaldehyde for 10 min and stained with 1 µg/ml DAPI in PBS/0.1% v/v Triton for another 10 min. Four fields per well were imaged using an IN Cell Analyzer 1000 (GE Healthcare, Buckinghamshire, UK) with a 4× objective and nuclei were counted by Investigator software (GE Healthcare, Buckinghamshire, UK). All values were normalized to the control treated with vehicle, analyzed using GraphPad Prism 5.04, and IC_50_ values were calculated by non-linear regression (variable slope, four parameter, least squares fit).

### Cell Cycle Analysis

Neuroendocrine tumor cell lines were seeded in 12-well plates in growth medium, cultured overnight, and treated as indicated. At distinct time points, cells including their supernatant were harvested and fixated in 70% ethanol for at least 24 h at −20°C. For cell cycle analysis, samples were washed with PBS, stained with propidium iodide solution (20 µg/ml propidium iodide, 20 µg/ml RNaseA in PBS), and 10,000 events per sample were counted and analyzed using a FACSCalibur flow cytometer (Becton Dickinson, Franklin Lakes, NJ, USA).

### Internalization Assay and Immunofluorescence

Cells were grown on coverslips for at least 24 h. For internalization studies, they were incubated with or without 1 µM somatostatin-14 (Bachem, Bubendorf, Switzerland) or octreotide (peptides & elephants, Hennigsdorf, Germany) in serum-free medium for 30 min at 37°C. Cells were washed with PBS, fixated in 1:1 acetone/methanol for 2 min, and incubated with anti-SSTR2 primary antibody (sc365502, Santa Cruz Biotechnology, Dallas, TX, USA) diluted 1:50 in PBS overnight at 4°C. After washing with PBS, cells were incubated with a Cy3-conjugated goat anti-mouse secondary antibody (#115-165-146, Jackson ImmunoResearch, West Grove, PA, USA) diluted 1:1,000 in PBS for 1 h at room temperature. After washing with PBS, coverslips were briefly dipped into 96% ethanol, air-dried, and mounted on glass slides with Immu-Mount (Thermo Fisher Scientific, Waltham, MA, USA). Mounted cells were imaged using a confocal laser scanning microscope (LSM510, Carl Zeiss, Jena, Germany) using a helium–neon laser at 543 nm, LP560 emission filter, and 40× or 63× NeoFluar oil immersion objectives. RIN-1038-SSTR2-GFP cells were not immunostained after fixation, but otherwise treated the same. To visualize GFP fluorescence, an argon laser at 488 nm, LP505 emission filter was utilized on the same microscope.

### Radioligand Binding Assay

Peptide iodination and radioligand-binding assays were performed as previously described (21). Briefly, 10 nmol Tyr^11^-somatostatin-14 (Tyr^11^–SST14, Bachem, Bubendorf, Switzerland) were iodinated by the chloramin T method (22) with 1 mCi carrier-free Na^125^I (NEZ033L010MC, Perkin Elmer, Waltham, MA, USA) and purified by HPLC (Analytic HPLC 1200 Series, Agilent, Santa Clara, CA, USA) (see Figure S1 in Supplementary Material). For competitive radioligand binding assays, 40,000 cells were seeded in 96-well plates and grown overnight. The next day, cells were incubated in binding buffer (50 mM Hepes, pH 7.4, 5 mM MgCl_2_, 1 mM CaCl_2_, 0.5% w/v BSA, cOmplete protease inhibitors) containing 100,000 counts per minute (cpm) [^125^I]-Tyr^11^-SST14 and increasing concentrations of unlabeled peptide. After 30 min at 37°C, cells were washed with ice-cold washing buffer (50 mM Tris–HCl, pH 7.4, 125 mM NaCl, 0.05% w/v BSA), lysed with 1 N NaOH, transferred to vials, and measured in a gamma counter (Wallac 1470 Wizard, Perkin Elmer, Waltham, MA, USA). The obtained cpm values were analyzed with GraphPad Prism 5.04, and IC_50_ values were calculated by non-linear regression (one site—fit logIC_50_, least squares fit).

## Results

**Figure 1:**
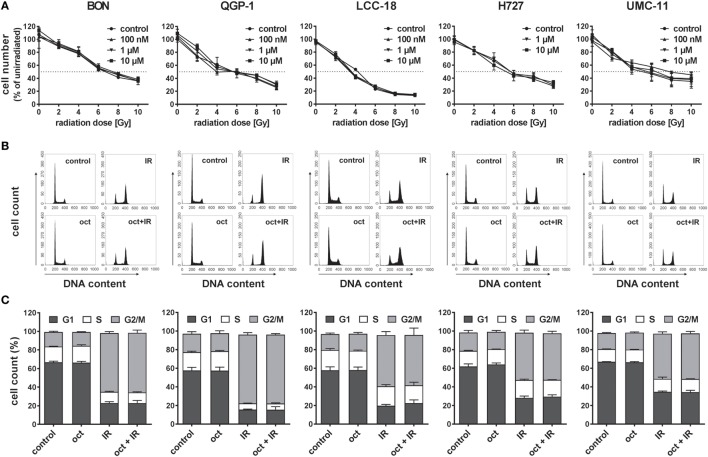
Pretreatment with octreotide does not modulate the impact of irradiation on neuroendocrine tumor (NET) cell lines. NET cell lines were incubated with 100 nM, 1 µM, or 10 µM octreotide or vehicle (control) for 24 h before irradiation. **(A)** Cell number was determined 96 h after irradiation with doses from 0 to 10 Gy. Graphs show mean ± SD of four replicates. **(B,C)** For assessment of cell cycle distribution, NET cells pretreated with vehicle (control) or 1 µM octreotide (oct) were collected 24 h after irradiation with 10 Gy (IR, oct + IR), stained with propidium iodide, and analyzed by flow cytometry. Data are shown as DNA histograms of one representative experiment **(B)** or as bar diagrams with mean ± SEM (*n* = 2–3) **(C)**.

In order to evaluate the influence of the SSA octreotide on the antiproliferative effect of radiation, five established NET cell lines from different organs of origin were studied: BON and QGP-1 cells from pancreatic NET, LCC-18 from a colon NET, as well as H727 and UMC-11 cells originating from pulmonary NETs. To ensure generating the maximum possible effect, all these cells were incubated with or without 100 nM, 1 µM, or 10 µM octreotide for 24 h before irradiating them with a single dose of 0–10 Gy using a ^137^Cs source. 96 h after irradiation, cells were stained using DAPI and nuclei were counted using a high-content analysis system. Results show a clear dose-dependent antiproliferative effect of radiation in all cell lines investigated, as cell numbers are increasingly reduced by increasing radiation doses (Figure 1A). The dose required to lower cell numbers in this assay by 50% was about 4 to 6 Gy for all cell lines. However, pretreatment with octreotide did not result in a measurable effect on cell number in any of these cell lines, at any concentration, as dose-response curves look nearly identical for all treatment protocols (Figure 1A). Likewise, when cell cycle distribution after treatment was investigated, all cell lines demonstrated a strong G2/M arrest 24 h after irradiation. While under control conditions the G2/M fraction was about 20% for all cell lines, after irradiation it was highest in QGP-1 (73.9 ± 2.1%) and BON (63.3 ± 1.6%) and strongly increased in LCC-18 (55.0 ± 3.8%), H727 (50.9 ± 2.8%), and UMC-11 (48.5 ± 2.0%) (Figures 1B,C). Pretreatment with 1 µM octreotide did not modulate these effects. Intriguingly, 1 µM octreotide alone also did not change cell cycle distribution as compared to control, raising the question whether these NET cell lines were responsive to treatment with SSAs.

**Figure 2:**
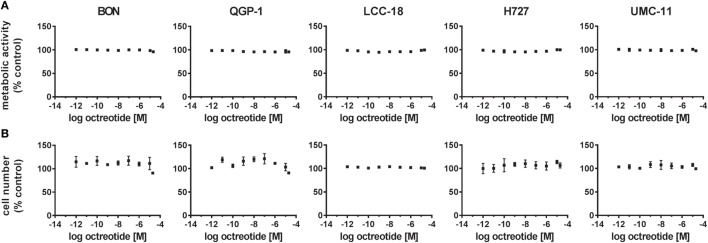
Octreotide treatment does not influence viability (metabolic activity) or proliferation (cell number) of neuroendocrine tumor (NET) cell lines. NET cell lines were treated with increasing concentrations of octreotide (0.001 nM–20 µM), incubated for 96 h and analyzed for metabolic activity **(A)** and cell number **(B)**. Data represent mean ± SEM (*n* = 2) as percent of control treated with vehicle.

Two assays were utilized to address this question: a viability assay measuring the metabolic activity, and a cell counting assay to describe proliferation or cell death by determining the number of nuclei. In both assays, increasing concentrations of octreotide did not lead to any change. Metabolic activity as well as cell number, given as percent of control, remained unaffected across the whole concentration range for all five NET cell lines, thereby confirming their lack of responsiveness to SSA treatment (Figure 2).

**Figure 3:**
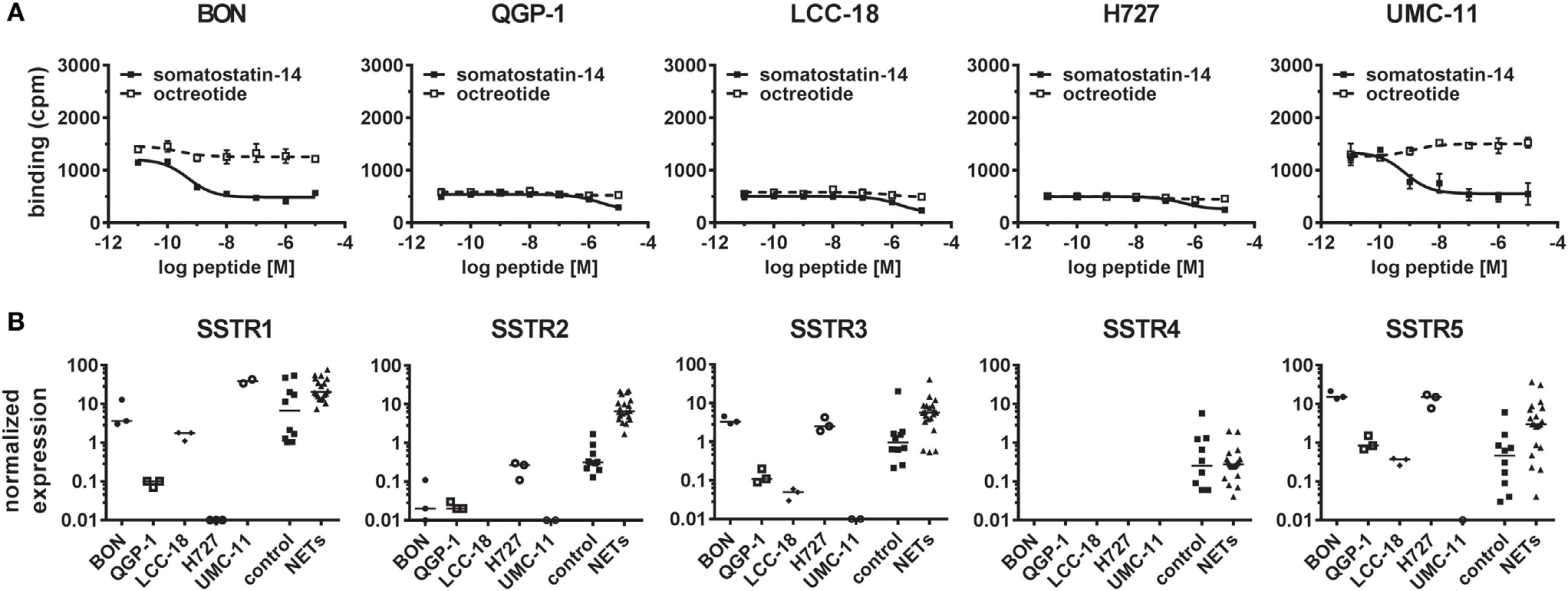
Radioligand binding assay and gene expression analysis reveal lack of SSTR2 expression in neuroendocrine tumor (NET) cell lines. **(A)** For competitive radioligand binding, NET cell lines were incubated with ^125^Iodine-labeled Tyr^11^–somatostatin-14 and increasing concentrations of unlabeled somatostatin-14 or octreotide (0.01 nM–10 µM). Data represent mean ± SD from duplicate measurements. **(B)** Scatter plots showing relative gene expression levels from reverse transcription quantitative real-time PCR of SSTR1-5 in NET cell lines (*n* = 3, different passages) in comparison to control tissues (*n* = 10) and NET tissues (*n* = 20). Values were normalized to ALG9 and HPRT1. Bars represent the median value.

As presentation of SSTR-binding sites at the cell surface is a prerequisite for functional response of NET cells to SSA treatment, a competitive radioligand binding assay was utilized to determine their binding capacity. While QGP-1, LCC-18, and H727 showed only background levels of [^125^I]-Tyr^11^-SST14 binding with no displacement by increasing amounts of either somatostatin-14 or octreotide, both BON and UMC-11 demonstrated binding above background that could be displaced by unlabeled somatostatin-14 (IC_50_ values: 0.50 ± 0.16 nM in BON and 0.70 ± 0.29 nM in UMC-11), but not by octreotide, indicating a lack of binding sites for octreotide in all five NET cell lines (Figure 3A). In order to examine whether this absence of binding was due to insufficient expression, all five SSTR subtypes were analyzed by RT–qPCR for their mRNA expression in the five NET cell lines under investigation and, for comparison, in 10 human normal tissues (5 from pancreas, 5 from small intestine) as well as 20 human NET tissues (10 from pancreatic NET, 10 from small intestinal NET). In all five cell lines, the expression of the receptor subtype with the highest affinity for SSAs used in the clinic, SSTR2, was at or below the level of expression in normal tissues and more than one order of magnitude lower than the median value of expression in NET tissues (Figure 3B). For BON, QGP-1, LCC-18, and UMC-11, SSTR2 expression was at least two orders of magnitude lower than in NET tissues. The receptor with the second highest affinity for clinically used SSAs, SSTR5, is also expressed at or below the median of normal tissues in QGP-1, LCC-18, and UMC-11, while in BON and H727, it is expressed at levels close to NET tissues. SSTR3 has moderate affinity for octreotide and other SSAs. It shows mRNA levels close to those of NET tissues in BON and H727, whereas the levels in the other cell lines were below the expression in normal tissues. SSTR1 was found to be highly expressed in UMC-11 (at the same level as in NET tissues), levels at or below normal tissues were detected in BON, LCC-18, and QGP-1. SSTR1 expression was lowest in H727. SSTR4 mRNA levels were similarly low in normal and NET tissues, and they could not be detected in any of the cell lines (Figure 3B).

**Figure 4:**
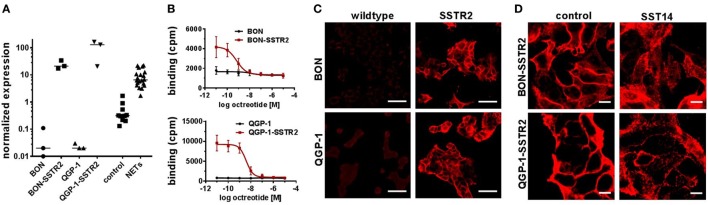
Validation of SSTR2 reintroduction into two neuroendocrine tumor (NET) cell lines. SSTR2 expression was confirmed by reverse transcription quantitative real-time PCR **(A)**, competitive radioligand binding assay **(B)** and immunofluorescence staining **(C,D)** in BON-SSTR2 and QGP-1-SSTR2 in comparison to wild-type BON and QGP-1 cells. **(A)** SSTR2 gene expression level in NET cell lines (*n* = 3, different passages) in comparison to control tissues (*n* = 10) and NET tissues (*n* = 20). Values were normalized to ALG9 and HPRT1. Bars represent median. **(B)** Cells were incubated with ^125^Iodine-labeled Tyr^11^-somatostatin-14 and increasing concentrations of octreotide (0.01 nM–10 µM). Data represent mean ± SEM (*n* = 2). **(C,D)** SSTR2–staining of BON-SSTR2 and QGP-1-SSTR2 in comparison to wild-type cells **(C)** or after incubation with or without 1 µM somatostatin-14 **(D)**. Scale bars represent 50 µm **(C)** or 10 µm **(D)**.

In summary, no octreotide binding was detected (Figure 3A) in any of the cell lines and mRNA levels for the preferred target of octreotide, SSTR2, were found to be at least 10-fold and up to 1,000-fold lower in NET cell lines than in human NET tissues (Figure 3B). Therefore, the lack of functional responses seen in viability, proliferation, and cell cycle analysis assays (Figures 1A–C and 2A,B) seemed likely to have its cause in low levels of SSTR2 expression in these cell lines. In order to rescue octreotide sensitivity, in two of the cell lines (BON, QGP-1) SSTR2 was reintroduced by stable transfection with a plasmid encoding human SSTR2. The success of SSTR2 re-expression was tested by RT-qPCR, radioligand binding assay, immunofluorescence, and internalization assay (Figures 4A–D). Quantification of human SSTR2 mRNA by RT-qPCR demonstrated successful expression of the target in both BON-SSTR2 and QGP-1-SSTR2 cells, with mRNA levels about 1,000-fold higher than in wild-type BON and QGP-1 and similar to mRNA levels found in human NET tissues (Figure 4A). Consequently, considerably higher binding levels were detected in the [^125^I]-Tyr^11^-SST14 radioligand binding assay in transfected cells as compared to wild-type cells: 2.5-fold higher in BON-SSTR2 and 9-fold higher in QGP-1-SSTR2 (Figure 4B). Importantly, this binding could be completely displaced by increasing concentrations of unlabeled octreotide at IC_50_ values in the expected subnanomolar to low nanomolar range (0.67 ± 0.32 nM for BON-SSTR2, 3.62 ± 0.23 nM for QGP-1-SSTR2). Similarly, immunofluorescence staining using a monoclonal antibody specific for human SSTR2 in wild-type BON and QGP-1 cells yielded only faint signals, whereas after reintroduction of SSTR2 into these cell lines, a clear staining signal was observed (Figure 4C). The distribution of SSTR2 staining suggested a predominant localization of the receptor at the plasma membrane. Taken together, SSTR2 detection in transfected cells using RT-qPCR, radioligand binding, and immunofluorescence staining strongly suggested successful SSTR2 reintroduction into BON and QGP-1 cells, thereby leading to sufficient levels of plasma membrane receptors. To verify whether these receptors would also show functional activity, a microscopic internalization assay was performed. BON-SSTR2 and QGP-1-SSTR2 were incubated for 30 min at 37°C under control conditions (medium only) or with 1 µM somatostatin-14 in medium. Controls showed predominantly plasma membrane localization as seen before, whereas after incubation with the agonist, a more diffuse, punctate pattern was observed, indicating a functional, ligand-dependent SSTR2 internalization into an intracellular vesicle compartment (Figure 4D). Similarly, cells were incubated with or without octreotide to principally demonstrate that there is downstream activity of octreotide in SSTR2-transfected BON and QGP-1 cells. As data in Figure S2 in Supplementary Material shows, octreotide has a profound effect on SSTR2 distribution in these cells (as well as in rat RIN-1038 cells transfected with a ratSSTR2-GFP plasmid). In the absence of ligand, the receptor localizes at the plasma membrane. Upon ligand addition, a translocation to an intracellular compartment becomes clearly visible (Figure S2 in Supplementary Material).

**Figure 5:**
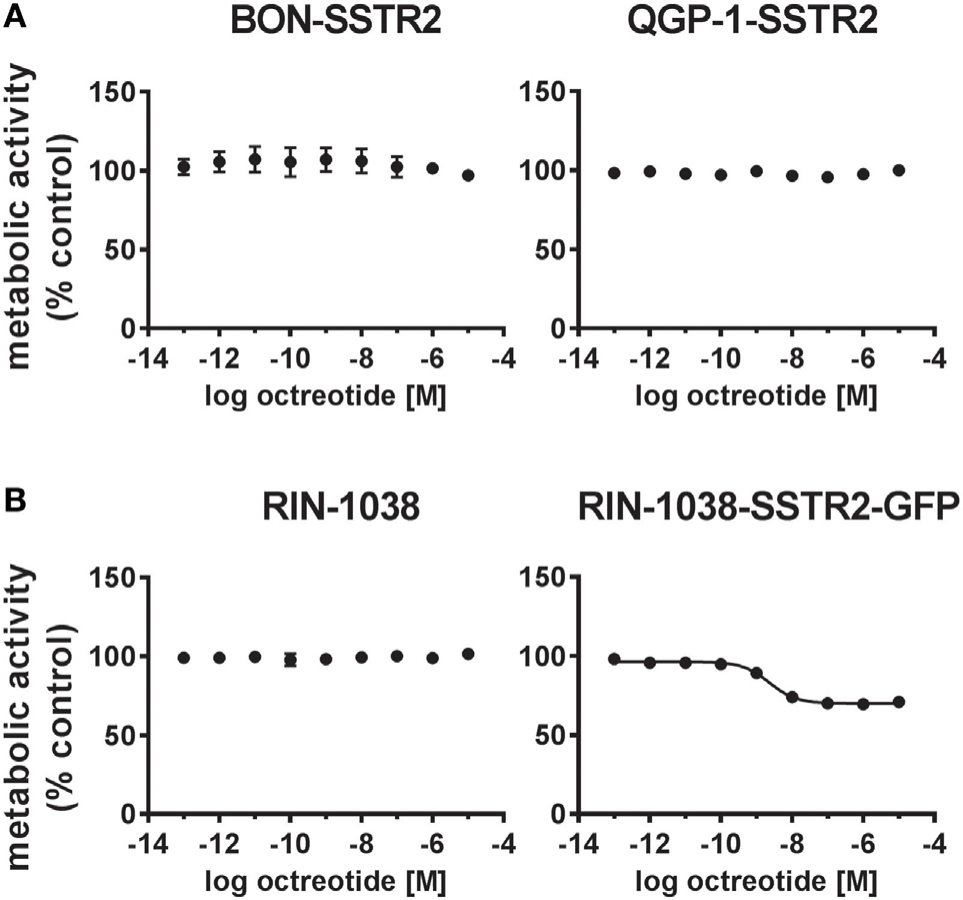
SSTR2 reintroduction does not sensitize human neuroendocrine tumor (NET) cells to octreotide treatment. **(A)** Human SSTR2-transfected NET cell lines BON and QGP-1 were treated with increasing concentrations of octreotide (0.1 pM–10 µM), incubated for 96 h and analyzed for viability (metabolic activity). **(B)** For comparison, wild-type and ratSSTR2-GFP-transfected RIN-1038 cells were analyzed in the same way. Data represent mean ± SEM (*n* = 2–4).

After demonstrating expression at NET-like levels and functional activation of the transfected SSTR2 in both cell lines, the functional consequences for viability of BON-SSTR2 and QGP-1-SSTR2 under SSA treatment were investigated. Both transfected cell lines were incubated with increasing concentrations of octreotide (0.1 pM–10 µM). However, no activity of the SSTR2 agonist was detected in the viability assay (Figure 5A). To evaluate whether re-expression of SSTR2 in another cell line would be able to sensitize cells to octreotide, we overexpressed the GFP-tagged rat SSTR2 in the rat insulinoma cell line RIN-1038. When both wild-type RIN-1038 and RIN-1038-SSTR2-GFP were subjected to treatment with increasing concentrations of octreotide, a difference was observed: while wild-type cells showed no octreotide response in viability, RIN-1038-SSTR2-GFP showed sensitivity toward octreotide with an IC_50_ of 2.29 ± 2.23 nM and an intrinsic activity of 31% (Figure 5B).

## Discussion

Peptide receptor radionuclide therapy using peptides such as DOTATOC and DOTATATE has evolved to be a major treatment option in the management of NETs and is associated mostly with partial and minor remission with objective response rates ranging from 4 to 30% (17). While previous PRRT studies were typically performed as monocentric retrospective studies, the prospective multicenter NETTER-1 trial (PRRT plus SSA vs. SSA alone) has recently demonstrated similar results with regard to response rate and progression-free survival, with overall survival at interim analysis appearing very encouraging (18). However, in other therapeutic settings, nuclear therapies perform even better: in metastasized differentiated thyroid cancers, radioiodine therapy can achieve a complete remission in at least a third of patients (23). Radioimmunotherapy with anti-CD20 antibodies in B-cell non-Hodgkin’s lymphoma achieves complete responses in 75% of patients (24). In stark contrast, in PRRT using SSAs, complete remission is extremely rare despite the fact that a radiation dose of as high as 250 Gy can be delivered to the tumors (25). Other established NET therapies such as SSA treatment may have a positive or negative impact on response rates by either radiosensitizing or radioprotecting tumors before PRRT. Therefore, the initial experiments in this study were designed to evaluate a potential synergistic or antagonistic effect of combined radiation and octreotide treatment in five established NET cell lines. To represent a broad range of NET subtypes, well-established cell line NET models from pancreas (BON, QGP-1), colon (LCC-18), and lung (H727, UMC-11) were included.

Treatment with a ^137^Cs source yielded a measure for the *in vitro* sensitivity of the cells toward ionizing radiation: the dose required to diminish cell number by 50% of control was found to be in a close range, between 4 and 6 Gy. However, octreotide applied as a pretreatment did not alter cell number or cell cycle distribution in any of the cell lines (Figure 1), indicating a lack of radiosensitization by the drug. External gamma radiation delivery has been chosen here, because it delivers reproducible, stable, and precise dose levels to cells in culture. Yet, the results obtained may not be fully predictive of PRRT using beta emitters as the latter exhibits differences in the nature of radiation, its energy spectrum, and kinetics. Radiopharmaceutical treatment will also depend on more complex phenomena such as biodistribution and tissue clearance kinetics, all potentially leading to differences in DNA profiles as well as damage recognition and repair mechanisms.

To verify whether SSA treatment alone would have an impact on viability or proliferation, cells were submitted to octreotide treatment up to a concentration of 20 µM. However, the drug did neither influence metabolic activity nor cell number (Figure 2). Direct and indirect antitumor effects of somatostatin and SSAs *via* SSTRs have so far been studied mostly in overexpression systems involving non-neuroendocrine, in part non-human cell lines such as CHO, COS, and HEK293 (8). Studies demonstrating a clear antiproliferative effect of SSAs in human neuroendocrine cell lines *in vitro* are scarce—mostly the impact was observed at concentrations of 1–10 μM, several orders of magnitude above published EC_50_ values for SSAs on SSTRs (0.2–10 nM) (7, 26, 27). One potential cause for the octreotide resistance of the five cell lines under investigation in this study may be a lack of target expression. Indeed, radioligand binding experiments and gene expression analysis demonstrated low binding and expression of SSTRs (Figure 3). BON and UMC-11 cells showed some binding; however, this could not be displaced by octreotide (Figure 3A). Direct comparison of SSTR mRNA levels in these cell lines with levels in normal human tissue and human NET tissues showed considerably lower expression of most SSTRs in the cell lines than in the tumors. For the most important target of octreotide, SSTR2, mRNA levels were at least one, typically three orders of magnitude lower in the cell lines than in human tumors. These results strongly suggested that a lack of target expression, particularly of SSTR2, might have caused the octreotide resistance observed in these cell lines. SSTR mRNA expression in NET cell lines has been studied before (28–30). To the best of our knowledge, this is the first study to directly compare expression levels in NET cell lines as well as human normal and NET tissues in a quantitative manner.

In order to verify the hypothesis that re-expression of SSTR2 may rescue octreotide sensitivity, two of the cell lines were transfected with a plasmid encoding human SSTR2. Successful re-expression in BON and QGP-1 cells at levels similar to those found in human NET tissues led to functional cell surface expression, as confirmed by octreotide-induced radioligand displacement, immunofluorescence, and ligand-induced receptor internalization (Figure 4). However, viability of these transfected cells under octreotide treatment up to 10 µM was still unaffected, indicating there must be further causes for this SSA resistance (Figure 5). A partial response with single-digit nanomolar IC_50_ was observed in RIN-1038 rat insulinoma cells transfected with rat SSTR2-GFP yet not in the parental wild-type cells, demonstrating the principle feasibility of this approach in a neuroendocrine cell line. This, in conjunction with the subnanomolar to low nanomolar IC_50_ values obtained in the radioligand binding assays (Figure 4B) and with the octreotide activity seen in the internalization assay (Figure S2 in Supplementary Material), provides solid evidence for the integrity and potency of the octreotide used here. However, two human NET cell lines could not be resensitized to SSA treatment by re-establishing their SSTR2 expression. Obviously, other mechanisms such as a lack in one or more signal transduction components that would relay a signal from the receptor to the cell cycle machinery are in place in BON and QGP-1 cells, preventing them from responding to octreotide. The signal was shown to be relayed at least on to the β-arrestin/dynamin-associated vesicle formation and internalization machinery in both SSTR2-transfected cell lines, as otherwise the translocation of the receptor to an intracellular vesicle compartment (Figure S2 in Supplementary Material; Figure 4D) would not occur. Antiproliferative and proapoptotic action of SSAs has been proposed to be transduced, e.g., *via* tyrosine phosphatases and ERK/Akt signaling (8). It is intriguing to speculate that signaling components essential for these pathways may have been lost in these cells or may never have been part of these cells’ inventory. There is no evidence yet from clinical studies that antiproliferative SSA action directly targets tumor cells rather than endothelial, immune or other cells of the tumor microenvironment. At least two large clinical trials have demonstrated antiproliferative effects of SSAs in NET patients (4, 5). As evidence for a direct action of SSAs on tumor cells *in vivo* is not available, a different mode of action should be considered, specifically on cells in the tumor microenvironment. The contribution of drug effects on cancer-associated fibroblasts, endothelial cells, macrophages, and other immune cells has been discussed as an antitumor mechanism in NETs and other tumor entities (31–35).

The lack of relevant SSTR expression levels in all five cell lines as well as ras and p53 mutations identified in some of them untypical for NET (36) call into question the suitability of these and other established NET cell lines as models to study regulation by SSAs (and potentially other drugs). In this set of experiments, different passages, including the earliest available for the respective cell line, were studied. Yet, no differences were identified in SSTR expression levels (see low variance in Figure 3B). Still, receptor downregulation and other perturbations may have occurred early on during the establishment of these cells lines and their adaptation from an *in vivo* tumor to 2D culture on plastic with artificial media and fetal calf serum, in the absence of an authentic microenvironment. During the past 40 years, work with continuously growing cell lines has greatly contributed to basic as well as translational cancer research. However, attention should be given in choosing the optimal *in vitro* model for the respective scientific question and analytical approach. Continuous NET cell lines frequently seem to lack SSTRs: for two novel large cell NET cell lines, SSTRs could not be detected by immunohistochemistry (37). On the other hand, two out of three proposed midgut NET cell lines reported by Pfragner et al. responded well to octreotide treatment (38, 39). Similarly, midgut NET cell lines KRJ and GOT1 seem to express SSTRs at functional levels (40, 41). However, three of the aforementioned cell lines (KRJ, H-STS, and L-STS) were recently characterized as non-neuroendocrine, EBV-positive lymphoblastoid cells (42). NET cell lines of pancreatic origin with authentic SSTR expression were lacking until Benten et al. recently published the establishment of the first well-differentiated human pancreatic NET cell line (43). Primary cell cultures from NET tissue may represent more authentic, relevant models and have proven valuable in the investigation of NET physiology in the past (44–47). Likewise, organoid and other 3D cultures may develop as an option to improve relevance and translatability of *in vitro* research. Furthermore, more bona fide *in vivo* models such as patient-derived xenografts may assist in verifying results from traditional NET cell lines (48).

## Ethics Statement

This study was carried out in accordance with the recommendations of the local ethics committee at Charité—Universitätsmedizin Berlin with written informed consent from all subjects. All subjects gave written informed consent in accordance with the Declaration of Helsinki. The protocol was approved by the local ethics committee at Charité—Universitätsmedizin Berlin.

## Author Contributions

CG and VP conceived the study; BW contributed scientific direction of the project; SE and CG performed experiments and analyzed data; CG wrote the draft manuscript; SE, VP, BW, and CG reviewed and/or edited the manuscript before submission.

## Conflict of Interest Statement

The authors declare that the research was conducted in the absence of any commercial or financial relationships that could be construed as a potential conflict of interest.
