# Supplementary material for: Octreotide Does Not Inhibit Proliferation in Five Neuroendocrine Tumor Cell Lines
Source: Front Endocrinol (Lausanne). 2018 Apr 6;9:146. doi: 10.3389/fendo.2018.00146 (PMC5897986; doi:10.3389/fendo.2018.00146)
Supplement: Supplementary file 1 [file image_1.PDF]

Supplementary Figure 1

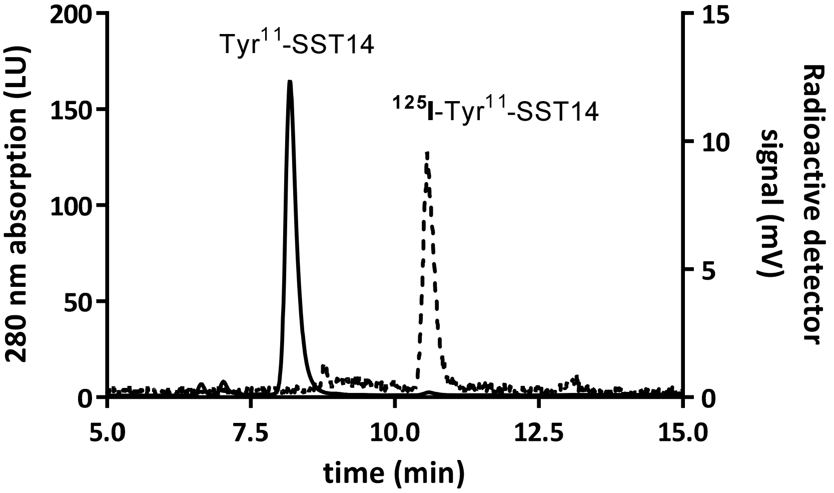

Supplementary Figure 1: HPLC purification of the radiolabeled tracer. For competitive radioligand binding experiments [^{125}I]-Tyr^{11}-somatostatin-14 was generated as described and purified via HPLC. [^{125}I]-labeled Tyr^{11}-somatostatin-14 showed a clear difference in retention time from unlabeled Tyr^{11}-somatostatin-14 and could be separated efficiently.
