# Supplementary material for: Octreotide Does Not Inhibit Proliferation in Five Neuroendocrine Tumor Cell Lines
Source: Front Endocrinol (Lausanne). 2018 Apr 6;9:146. doi: 10.3389/fendo.2018.00146 (PMC5897986; doi:10.3389/fendo.2018.00146)
Supplement: Supplementary file 2 [file image_2.PDF]

Supplementary Figure 2

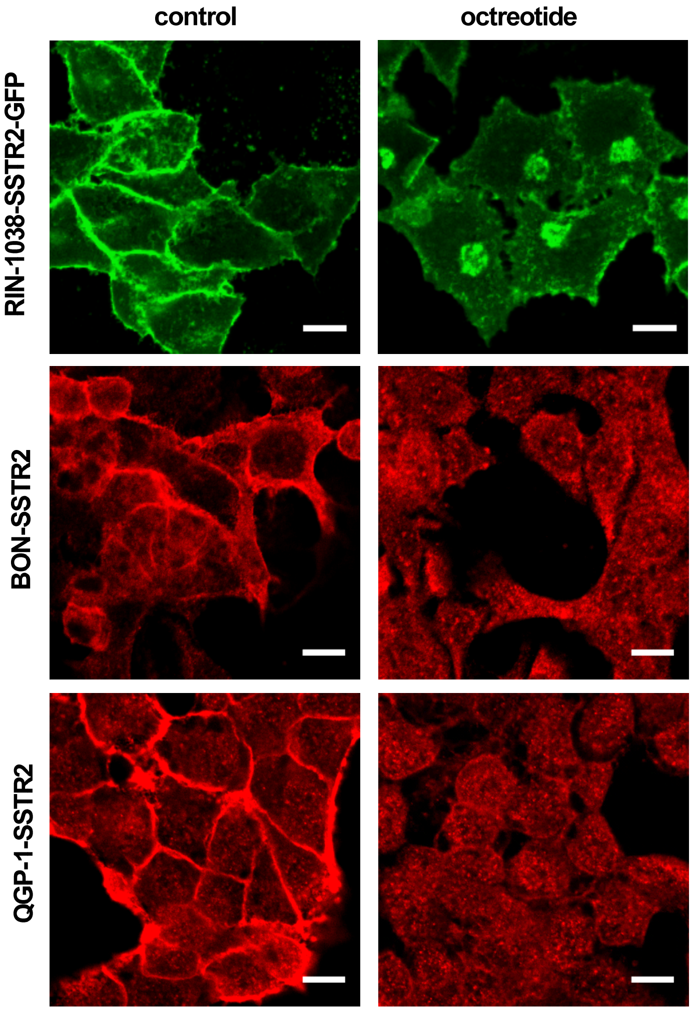

Supplementary Figure 2: Octreotide activity in an internalization assay. In order to provide evidence for octreotide activity, RIN-1038-SSTR2-GFP, BON-SSTR2 and QGP-1-SSTR2 cells were incubated in the absence (control) or presence (octreotide) of 1 µM octreotide for 30 minutes before fixation, immunostaining of SSTR2 (except for RIN-1038-SSTR2-GFP), mounting and analysis using a confocal laser-scanning microscope. Octreotide leads to a profound redistribution of the receptor: in its absence, SSTR2 is localized mainly to the plasma membrane. In the presence of the ligand, the receptor almost completely translocates to an intracellular compartment which appears to be perinuclear, TGN-associated in RIN-1038-SSTR2-GFP cells while more diffusely vesicular in the two transfected human cell lines BON-SSTR2 and QGP-1-SSTR2. Scale bars represent 10 µm.
