# Supplementary material for: Octreotide Does Not Inhibit Proliferation in Five Neuroendocrine Tumor Cell Lines
Source: Front Endocrinol (Lausanne). 2018 Apr 6;9:146. doi: 10.3389/fendo.2018.00146 (PMC5897986; doi:10.3389/fendo.2018.00146)
Supplement: Supplementary file 3 [file table_1.PDF]

**Supplementary Table 1**

Forward and reverse primer sequences used for RT-qPCR.

| Gene | Forward primer sequence | Reverse primer sequence | Length (bp) |
|---|---|---|---|
| SSTR1 | TGAGTCAGCTGTCGGTCATC | ACACTGTAGGCACGGCTCTT | 184 |
| SSTR2 | CCCCTCACCATCATCTGTCT | AGGTGAGGACCACCACAAAG | 247 |
| SSTR3 | TGCTCAACATCGTCAACGTG | TAAAGGATGGGGTTGGCACAG | 115 |
| SSTR4 | TCAACCACGTGTCCCTTATCC | AAGAATCGGCGGAAGTTGTC | 91 |
| SSTR5 | TCACCGTCAACATCGTCAAC | TGGCGGAAGTTGTCAGAGAG | 145 |
| HPRT1 | TGACACTGGCAAAACAATGCA | GGTCCTTTTCACCAGCAAGCT | 94 |
| ALG9 | GTCTTCTGGCTTTTGTGAGCTG | TCACGTGCAACCCAAACTTC | 78 |
