# Supplementary material for: Octreotide Does Not Inhibit Proliferation in Five Neuroendocrine Tumor Cell Lines
Source: Front Endocrinol (Lausanne). 2018 Apr 6;9:146. doi: 10.3389/fendo.2018.00146 (PMC5897986; doi:10.3389/fendo.2018.00146)
Supplement: Supplementary file 4 [file table_2.PDF]

Supplementary Table 2

Clinical and histochemical characteristics of the human tissues analyzed.

ID	sex	age at surgery	diagnosis	location of primary	tissue type	organ	Ki67 [%]	grade
16	F	59	NET	pancreas	metastasis	lymph node met	5	G2
21	M	61	NET	pancreas	metastasis	liver met	35	G3
39	M	36	gastrinoma	pancreas	primary	pancreas	1	G1
12	F	24	NET	pancreas	primary	pancreas	5	G1
1	M	67	NET	pancreas	metastasis	liver met	3	G2
10	F	64	insulinoma	pancreas	primary	pancreas	5	G2
26	M	63	NET	pancreas	metastasis	liver met	90	G3
39	M	36	gastrinoma	pancreas	primary	pancreas	2	G1
55	M	44	NET	pancreas	metastasis	liver met	2	G1
32	F	54	NET	pancreas	primary	pancreas	10	G2
N19	M	32		pancreas	normal			
N21	M	50		pancreas	normal			
N22	M	62		pancreas	normal			
AIN	M	n/a		pancreas	normal			
7A12	M	72		pancreas	normal			
58	M	35	gastrinoma	Ileal	primary	ileum	10	G2
61	M	58	NET	Ileal	metastasis	liver	2	G1
14	F	62	NET	pancreas	metastasis	liver	2	G1
23	M	71	NET	Ileal	primary	ileum	2	G1
8	F	74	NET	Ileal	primary	ileum	1	G3
15	F	49	NET	Ileal	primary	ileum	1	G1
10	F	56	NET	Ileal	metastasis	lymph node met	5	G2
79	F	55	NET	Ileal	primary	ileum	3	G2
42	F	71	NET	Ileal	primary	ileum	6	G2
46	F	54	NET	Ileal	primary	ileum	10	G2
80	M	35		Ileal	normal			
77	F	65		Ileal	normal			
62	F	70		Ileal	normal			
28	M	66		Ileal	normal			
53	M	69		Ileal	normal			
